# MSF-Net: Multi-Scale Feature Learning Network for Classification of Surface Defects of Multifarious Sizes

**DOI:** 10.3390/s21155125

**Published:** 2021-07-29

**Authors:** Pengcheng Xu, Zhongyuan Guo, Lei Liang, Xiaohang Xu

**Affiliations:** 1 College of Computer Science and Technology, Wuhan University of Technology, Wuhan 430070, China; xuguduxia@163.com (P.X.); L30L30@126.com (L.L.); 2 Radar Non Commissioned Officer School, Air Force Early Warning Academy, Wuhan 430019, China; 3 School of Electronic Information, Wuhan University, Wuhan 430072, China; guozhongyuan@whu.edu.cn

**Keywords:** surface defect classification, deep learning, convolutional neural network, multi-scale features, multi-size defects

## Abstract

In the field of surface defect detection, the scale difference of product surface defects is often huge. The existing defect detection methods based on Convolutional Neural Networks (CNNs) are more inclined to express macro and abstract features, and the ability to express local and small defects is insufficient, resulting in an imbalance of feature expression capabilities. In this paper, a Multi-Scale Feature Learning Network (MSF-Net) based on Dual Module Feature (DMF) extractor is proposed. DMF extractor is mainly composed of optimized Concatenated Rectified Linear Units (CReLUs) and optimized Inception feature extraction modules, which increases the diversity of feature receptive fields while reducing the amount of calculation; the feature maps of the middle layer with different sizes of receptive fields are merged to increase the richness of the receptive fields of the last layer of feature maps; the residual shortcut connections, batch normalization layer and average pooling layer are used to replace the fully connected layer to improve training efficiency, and make the multi-scale feature learning ability more balanced at the same time. Two representative multi-scale defect data sets are used for experiments, and the experimental results verify the advancement and effectiveness of the proposed MSF-Net in the detection of surface defects with multi-scale features.

## 1. Introduction

With the rapid development of the manufacturing industry, people are paying more and more attention to the surface quality of various industrial products. The surface quality of the product will not only affect the appearance and visual effect of the product, but also affect the internal quality and performance of the product. In order to reduce production costs, improve production efficiency and product quality, it is very necessary to effectively detect surface defects in the product manufacturing process. 

At present, the commonly used surface defect detection methods are as follows [1]:

1. Artificial visual inspection, which has the disadvantages of low detection efficiency, high false detection rate and high missed detection rate, high labor intensity, and low speed.
2. The non-contact detection method based on machine vision [2,3] usually adopts image processing algorithms or manual design feature extractors to combine the classifier. Liu T.I. [4] proposed a fuzzy logic expert system for roller bearing defect detection, the system combines frequency response and fuzzy reasoning and has achieved good results. Baygin et al. [5] used Otsu thresholding and Hough transform to extract features from the reference image for the problem of printed circuit board with defects and matched the image to be inspected with the reference image to accurately detect the missing holes on the circuit board. Zhang Lei et al. [6] proposed a fabric defect classification algorithm combining Local Binary Pattern (LBP) and Gray Level Co-occurrence Matrix (GLCM). The algorithm first uses the LBP algorithm to extract the local feature information of the image and then uses the GLCM to describe the overall texture information, and finally, the feature information of the two parts as a whole constructed as the input of the BP neural network, and a higher classification accuracy is obtained. Denis Sidorov et al. [7] proposed an automatic defect classification method based on the p-median clustering technique, the proposed method uses the p-median combinatorial optimization problem to complete the clustering problem, which can be sued in semiconductor and other manufacturing industries. In general, compared with artificial visual inspection methods, the above methods have the advantages of safety and reliability, high detection accuracy, and long-term operation in complex production environments, which effectively improves production efficiency and quality inspection efficiency. However, in a real and complex industrial environment, there are generally small differences between surface defects and background, low contrast, large differences in defect scales, and various types of defects. The design of image processing algorithm schemes and artificially designed feature extraction schemes typically requires rich expert experience and a large number of experiments, resulting in high cost and time consumption, and the effectiveness and generalization cannot be guaranteed, and it is difficult to obtain better detection results.

In recent years, with the successful application of deep learning models represented by convolutional neural networks (CNNs) [8] in computer vision fields such as face recognition [9], scene text detection [10], target tracking, and autonomous driving [11], the surface defect detection methods based on deep learning have also been widely used in various industrial scenarios and have become the mainstream method in the field of defect detection. Weimer [12] explored the influence of the design of CNN and different hyper-parameters on the accuracy of defect detection results. Ren [13] built a classifier based on the features of image patches, transferred the features from the pre-trained deep learning model, and convolved the trained classifier on the input image to obtain pixel-level predictions, compared with multi-layer perception and support vector machine, its error rate is lower. Masci [14] proposed a max-pooling CNN method for steel defect classification, experiments were performed on seven types of defects, the accuracy rate reached 93%, and its performance is far better than SVM classification trained on feature descriptors. Aiming at the problem of jujube surface defect detection, Guo [15] has done a series of work on data preprocessing, data augmentation, and composite convolutional neural network design, and achieved good results. Deitsch [16] used a modified VGG 19 network to identify solar panel image defects with a resolution of 300 × 300, with an accuracy rate of 88.42%, which exceeds a variety of manual design features and supported vector machine methods. Xu [17] presented a small data-driven convolutional neural network (SDD-CNN) to detect the subtle defects of rollers, the method first used label dilation to solve the problem of the imbalance of the number of classes, then a semi-supervised data augmentation method is proposed, and finally, CNNs were trained, experimental results show that compared with the original CNNs, SDD-CNNs has significantly improved the convergence time and classification accuracy. In addition, some advanced CNN structures have also achieved good detection results, including but not limited to references [18,19,20,21,22,23]. 

CNNs are currently used by domestic and foreign researchers and engineers as the preferred architecture for product surface defect classification. However, difficulties and challenges still exist. 

For different types of products, the surface defects often have the characteristics of different sizes, uncertain positions, and different shapes; even for the same type of product, the color, texture, shape, and size between different types of defects is also very different. The general CNN often contains a few specific scales of receptive fields and is more inclined to express macroscopic and abstract features. It is not strong in expressing local and small defects, which leads to an imbalance in feature expression capabilities. Therefore, how to design a deep CNN that can simultaneously take into account multi-scale feature extraction has become the focus of research.

Since the receptive fields of the convolution kernels in the CNN are closely related to the sizes of the target features, the CNN’s ability to express features at different scales directly determines its ability to detect defects of different sizes [24]. This paper starts with the analysis of the appearance and size characteristics of product surface defects, analyzes the processing mechanism of mainstream CNNs for different scale features, and tries to improve the expression and classification capabilities of deep CNNs for different scale features. On the basis of the above research, a Multi-Scale Feature Learning Network (MSF-Net) based on Dual Module Feature (DMF) extractor is proposed, experiments are carried out on two public multi-scale defect data sets, the experimental results verify the effectiveness and superiority of the MSF-Net proposed in this paper.

The structure of this paper is as follows. Section 2 introduces the related work. Section 3 is the research method. Section 4 is the experimental results and discussion, and Section 5 summarizes the paper. 

## 2. Related Work

**Figure 1:**
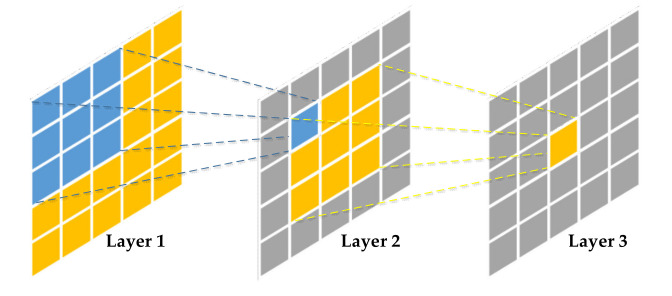
Schematic diagram of the receptive field in CNNs.

The receptive field refers to the input area that neurons can “see” in the CNN [25], as shown in Figure 1, the calculation of an element on the feature map in a CNN corresponds to a certain area on the input image, so the corresponding area is the receptive field of the element. It can be seen from Figure 1 that the receptive field is a relative concept, the elements on the feature map of a certain layer can see different areas on the previous layers. 

The receptive field $RF_{i}$ of the feature map of the i-th layer is shown by Formula (1):

(1) $$RF_{i}=RF_{i-1}+(K_{i}-1)*\overset{i-1}{\underset{k=1}{\prod }}S_{k}\;i\geq 1$$

where $K_{i}$ and $S_{i}$ respectively represent the size of the convolution kernel and the stride of the i-th convolution layer. In addition, for the input layer, $RF_{0}=1$, $S_{0}=1$.

In recent years, some researchers have tried to improve the classification performance of CNNs on multi-scale target data sets by optimizing the CNNs’ structure, such as the works of Tang [26] and Kim [27]. In essence, the optimization ideas for these works are derived from the classic CNN architecture, so the distribution of the receptive field in the classic CNNs are analyzed. 

### 2.1. AlexNet and VGGNet 

As we all know, there is no branch structure in the two CNNs of AlexNet [28] and VGGNet [29]. Therefore, the receptive field size of the last convolution layer before the fully connected layer is uniform, and the receptive field size is shown in Table 1. 

It can be seen from Table 1 that the size of the feature map output by the last convolutional layer of AlexNet is 195 × 195, while the size of the feature map output by the last convolutional layer of VGG-16 is 212 × 212, and the input image size of the two CNNs is 224 × 224. In other words, what the two CNNs finally extract are macroscopic and abstract features, and the extraction of tiny and concrete features is insufficient.

### 2.2. GoogLeNet and ResNet 

Unlike AlexNet and VGGNet, GoogLeNet [30] and ResNet [31] have rich branch structures. This is due to the modularization of the two CNNs, the smallest module unit of GoogLeNet is called the Inception module, and the smallest module unit of ResNet is the Residual Block. 

**Figure 2:**
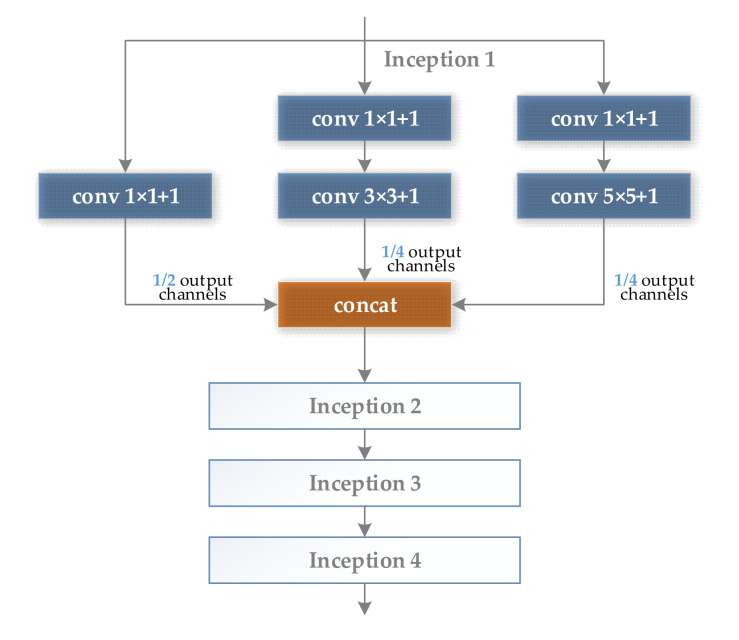
Four Inception modules in series structure.

**Figure 3:**
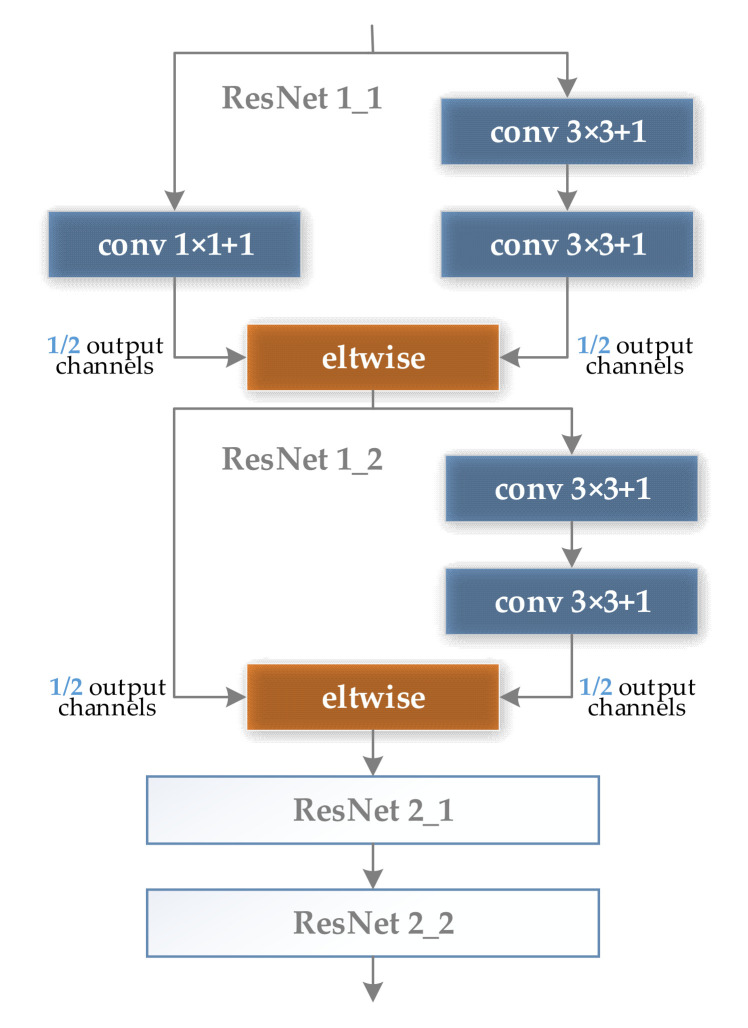
Two residual modules in series structure.

Figure 2 shows the structure of four simplified Inception modules connected in series, for the convenience of calculation, the ratio of the number of output feature maps of the three branches of 1 × 1, 3 × 3, and 5 × 5 is set to 2:1:1. Figure 3 shows the structure of two residual modules connected in series, and the output ratio of feature maps of all branches is set to 1:1.

**Figure 4:**
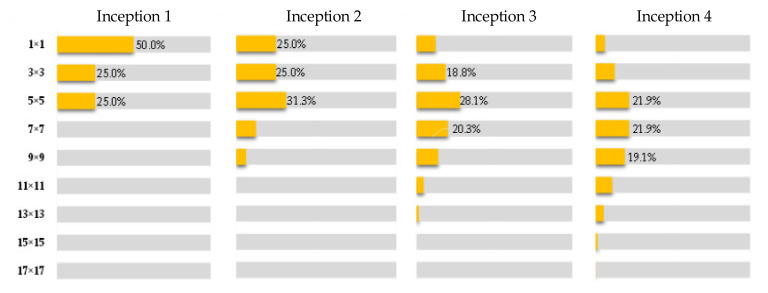
Distribution of the receptive field of the series structure of four Inception modules.

**Figure 5:**
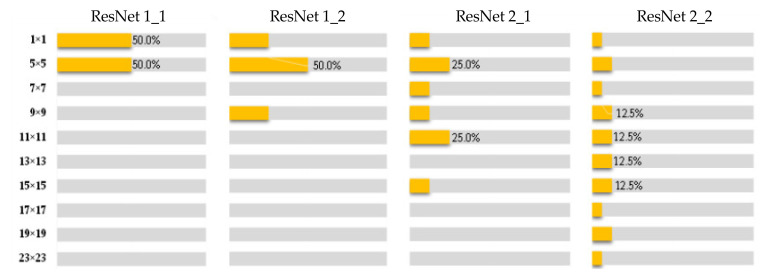
Distribution of the receptive of receptive field of the series structure of two residual blocks.

Figure 4 and Figure 5 respectively reveal the evolution of the receptive fields of the corresponding feature maps of the GoogLeNet and ResNet as the convolutional layer deepens. It can be seen that although the specific number of receptive fields at each scale is not completely the same, both of them reveal a common phenomenon. That is, in the initial stage of feature extraction by CNNs, the size of the receptive field is small, it is sensitive to the micro and local feature information of the image, and the learning ability is strong; in the middle and last stages of feature extraction by CNNs, as the number of convolution operations increases, the feature map becomes more abstract and is more inclined to express macro and global information. 

In addition, although in the distribution map of the fourth module in Figure 4 and Figure 5, three are still small receptive fields such as 1 × 1, 3 × 3, and 5 × 5, but as the convolutional neural network becomes deeper, these local receptive fields will gradually disappear. Table 2 shows the minimum and maximum receptive field scale distributions of the last layer of feature maps in the GoogLeNet and ResNet-18, it can be seen that the local receptive fields have disappeared and been replaced by macroscopic large and even super large receptive fields.

In summary, the existing CNNs have similar characteristics in design, that is, in the early stage of feature extraction of CNNs, they focus on learning the local and concrete information of images, and in the middle and later stages of feature extraction of CNNs, the macro, and abstract feature information are more likely to be learned. Of course, the above law is logical and effective in most classification and detection tasks, but for the surface defect data sets with multi-scale features studied in this paper, the above design ideas of CNNs are obviously not the optimal choice.

## 3. Methods

### 3.1. Data Set Preparation

**Figure 6:**
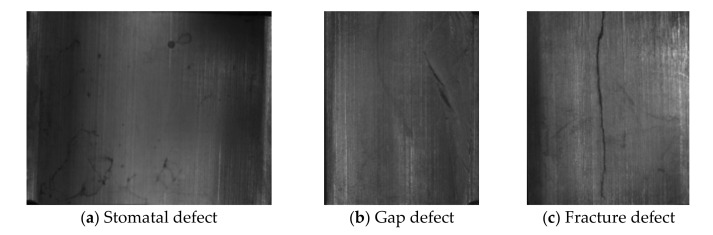

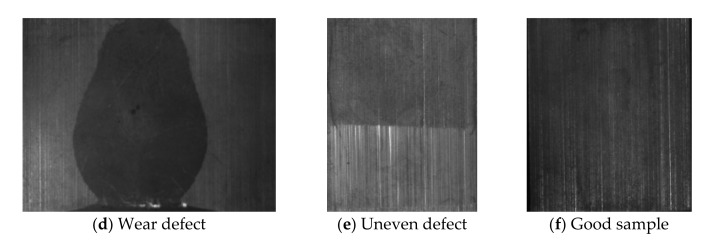
Samples of magnetic tile data set.

(1) The magnetic tile defect data set [17] was collected by the Institute of Automation of the Chinese Academy of Sciences, the data set has six categories, including non-defective samples, the representative samples of each category are shown in Figure 6. It should be noted that when using a CNN to train the magnetic tile data set, there is a problem with an insufficient number of samples, therefore, in this paper, the Semi-Supervised Data Augmentation (SSDA) method is used, the SSDA method takes into account the shape and location characteristics of the defective target on the basis of the classic data augmentation operation, and maintains the original label attributes of the samples while performing data augmentation, which provides high-quality data support for the training of CNNs [17], and finally get 10,320 sample images, including 6192 images in the training set, 2064 images in the validation set and 2064 image in the test set, the ratio of the training set, validation set and test set is 3:1:1.

(2) Roller surface defect data set [17], which is from the data set collected and published by the Institute of Automation of the Chinese Academy of Sciences (CAS). The roller surface defect data set collects various morphological samples of the roller surface in the air-conditioning compressor. The data set is made from the original image after preprocessing such as ring region expansion, sliding window cutting, image enhancement, etc. The sample examples and numbers of each category are shown in Table 3. Among them, EFQ and CQ are non-destructive surface samples, and the other categories are defective samples. After data augmentation, 22,400 samples are finally obtained, among them, there are 13,440 images in the training set, 4480 images in the validation set, and 4480 sheets in the test set, the ratio between them is also 3:1:1.

### 3.2. Sample Defect Size Analysis

**Figure 7:**
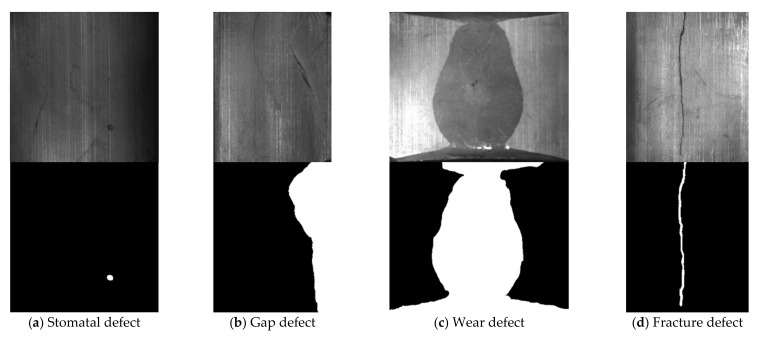
The samples with large scale difference of surface defects of magnetic tile and their corresponding defect labeling positions.

**Figure 8:**
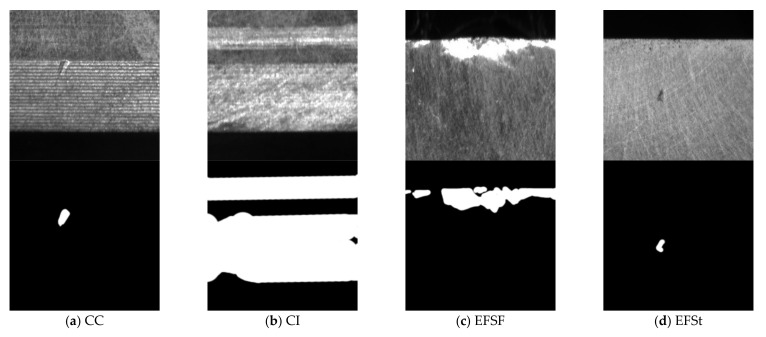
The samples with large scale difference of surface defects of roller and their corresponding defect labeling positions.

**Figure 9:**
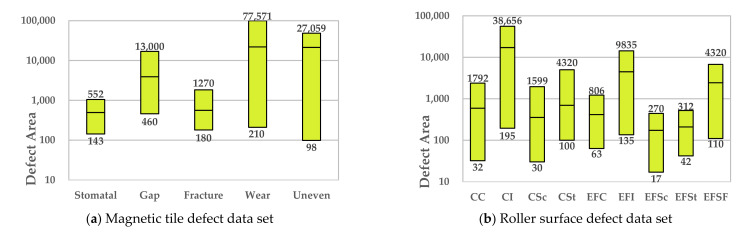
Defect area statistics of chart of different types of defect samples in the two data sets (The upper and lower lines of each column in the figure represent the maximum and minimum values respectively, and the middle line of each column represents the average value).

Figure 7 and Figure 8 respectively show samples with large differences in defect scales in the two datasets and their corresponding defect labeling positions, it can be seen that the size differences between different defect types are quite huge, which poses a great challenge to classification and recognition tasks. In addition, even for defects of the same type, the size of the defect varies from sample to sample. Figure 9 shows the area statistics of all defect types in the two datasets. It can be seen that in the defect data set of magnetic tile, the average area of wear defects and uneven defects is more than 60 times that of stomatal defects; the maximum defect area of the wear defect sample exceeds the maximum area of the stomatal defect sample by 140 times.

In the roller defect data set, the average defect area of defect samples of CI type is close to 17,000 pixels, while the average defect area of defect sample types such as CC, CSc, CSt, EFC, EFSc, EFSt do not exceed 1000. From the above analysis, it can be seen that these two defect data sets have obvious multi-scale defects problems.

### 3.3. Multi-Scale Feature Learning Network Based on Dual Module Feature Extractor

In order to solve the problem that the existing CNNs are not strong in learning small-scale defect features, and to improve the classification and recognize the ability of multi-scale surface defects, this paper proposes Multi-Scale Feature Learning Network (MSF-Net) based on Dual Module Feature Extractor (DMF), where DMF is built by the Concatenated Rectified Linear Units (CReLUs) and the Inception modules. The main design ideas of MSF-Net are as follows:

(1) Increase the diversity of the receptive field of a single convolution module in CNNs
  From the analysis of the receptive field of CNN in Section 2.1, it can be seen that single branch CNNs, such as AlexNet and VGGNet, have a relatively single receptive field scale. As the number of layers of the CNN deepens, the small-scale receptive field gradually disappears, which is not conducive to the feature expression of subtle defects in the classification task of surface defects.
  Therefore, this paper chooses the convolution module with branch structure as the basic unit of MSF-Net. Among the many representative modules with branch structures, the Inception module has favored domestic and foreign researchers in the field of target classification and detection, because of its lightweight design ideas and excellent characteristic expression ability and classification accuracy. In this paper, the Inception v3 [32] structure is used as the design prototype, as the feature extraction module in the middle and late stages of MSF-Net; and in the early stage of MSF-Net, in order to reduce the parameter quantity and calculation amount, this paper selects the CReLU module [33] as the prototype design feature extraction module. In this way, the Dual Module Feature (DMF) extractor is formed.
(2) Increase the diversity of the receptive field of the feature map output by the last convolutional layer of CNNs
  In order to improve the classification accuracy of multi-scale defect samples, it is necessary to ensure that the feature map output by the last convolutional layer before the fully connected layer has sufficient receptive field scales, especially the number of small-scale receptive fields. Therefore, inspired by HyperNet [34], the feature maps of several convolution modules with different scale receptive fields are combined to effectively increase the diversity of the receptive fields of the feature maps of the last convolution layer before the fully connected layer.
(3) Improve training efficiency
  The improvement of feature expression ability inevitably means the deepening of the number of layers of CNNs. Therefore, it is essential to improve training efficiency. MSF-Net improves training efficiency and avoids over-fitting by using residual shortcuts and batch normalization (BN) layers.

#### 3.3.1. Dual Module Feature Extractor

The Dual Module Feature Extractor (DMF) contains two different convolutional modules: the CReLU modules are mainly used in the early stage of feature extraction of MSF-Net, which aims to reduce the calculation cost and speed up the calculation of forward propagation; in the later stage of feature extraction of MSF-Net, the Inception modules are used to increase the depth and width of MSF-Net and improve feature learning ability.

##### Optimized CReLU Feature Extraction Modules

**Figure 10:**
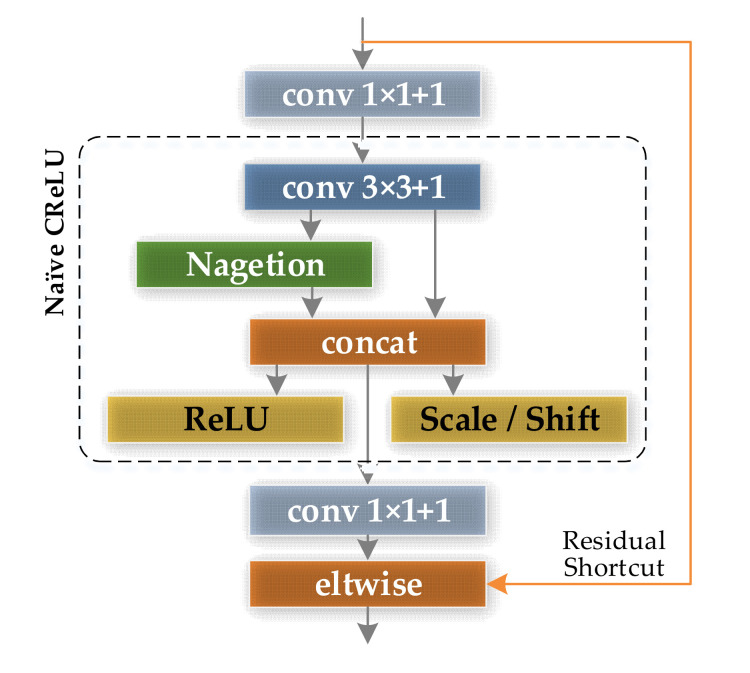
Optimized CReLU feature extraction module.

The research of Shang et al. [33] revealed that in the early stage of feature extraction of CNNs, the filters in the lower layers form pairs, the phase of each pair of filters is opposite, that is, CNN has a tendency to learn both positive and negative phase information at the same time, however, the Rectified Linear Units (ReLU) [35] will suppress the negative response, making the feature of the lower convolutional layer of the CNN redundant. CReLU takes these negative responses as the output of the convolutional layer by inverting the feature map and then using the ReLU function, the structure of CReLU is shown in the dashed box in Figure 10. The above operation can convert redundant features into usable features, and extract twice as many feature maps, thereby improving the utilization of features in the lower convolutional layer of CNN. In this paper, the CReLU feature extraction module is designed on the basis of the native CReLU module, as shown in Figure 10. A 1 × 1 convolutional layer is added to the input and output of the native ReLU module to achieve dimensionality reduction and dimensionality increase of the number of convolution kernel channels, which increases the nonlinearity of CNNs and reduces the amount of calculation. In addition, the shortcut connections in ResNet are also introduced into the CReLU feature extraction module to reduce the loss of information in the transmission process and protect the integrity of the information.

##### Optimized Inception V3 Module

The Inception V3 module uses convolution kernels of different sizes such as 1 × 1, 3 × 3, and 5 × 5 to obtain different scales of receptive fields, which improves the diversity of features. In addition, the design of the branch structure saves computational costs while enhancing the width and depth of CNN.

**Figure 11:**
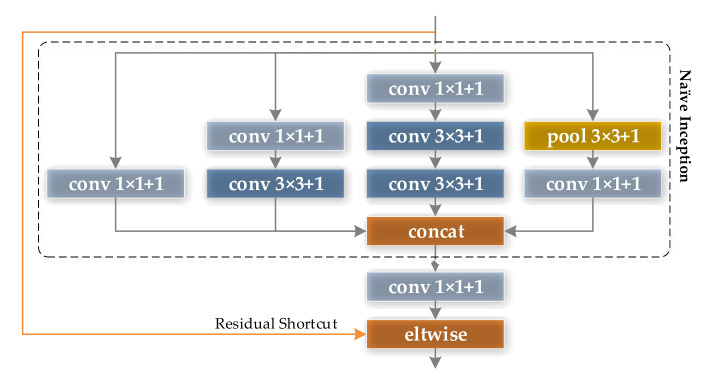
Optimized Inception feature extraction module.

Based on the native Inception V3 module, this paper proposed an optimized version of the Inception feature module, as shown in Figure 11. Similar to the optimized version of the ReLU module, a 1 × 1 convolutional layer is added to the output of the optimized module to realize the dimension increase of the feature output; in addition, shortcut connections are also introduced into the optimized Inception module.

#### 3.3.2. Multi-Scale Feature Learning Network

**Figure 12:**
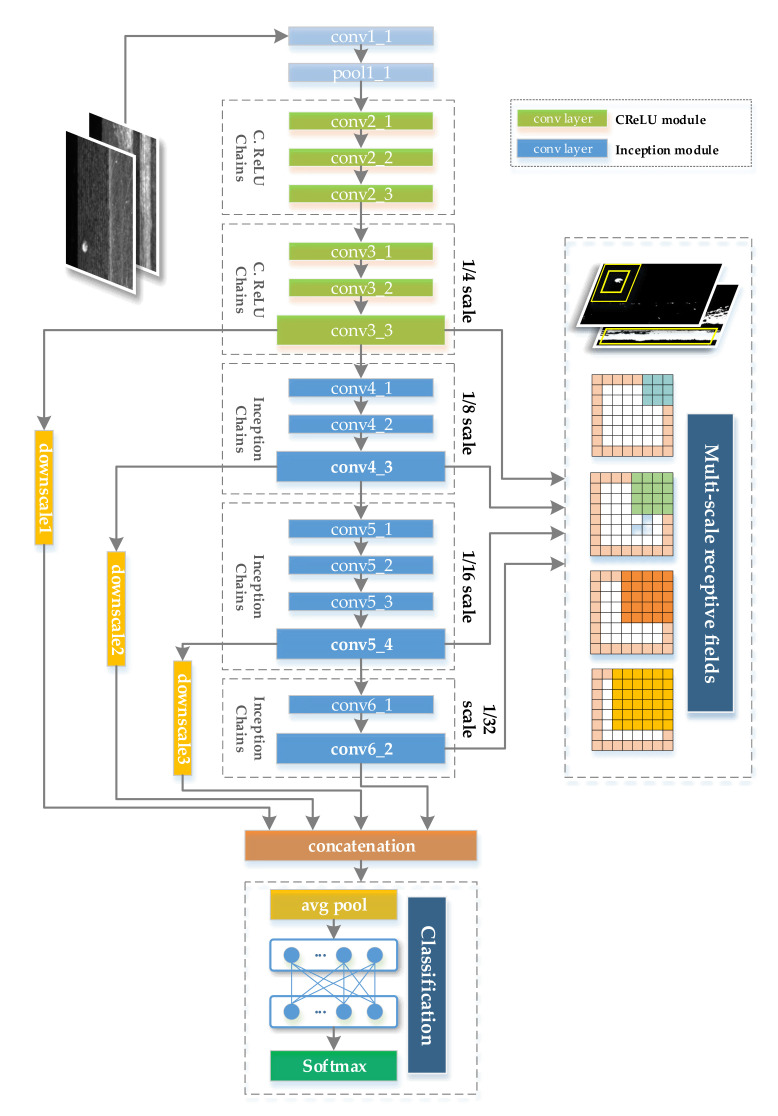
The overall architecture of MSF-Net.

In order to increase the diversity of the receptive field of the last convolutional layer of CNNs and improve the ability to learn and express features of different scales, especially small and local features, MSF-Net aligns and integrates the feature outputs of several intermediate layers, and then the fully connected layer is used for classification. Figure 12 shows the overall architecture of MSF-Net, and Table 4 lists the specific parameters and indicators in detail. As can be seen from Figure 12, MSF-Net is mainly composed of five convolutional module chains in the feature extraction stage, including two optimized CReLU modules and three optimized Inception V3 modules. In addition, all convolutional layers in MSF-Net, except for the optimized CReLU modules, are designed with batch normalization (BN) layers, scale layers, and ReLU activation layer to better accelerate convergence.

In order to realize the learning and expression of multi-scale features, the specific design details of MSF-Net are as follows:

(1) At the input of the convolution modules conv3_1, conv4_1, conv5_1, and conv6_1, the convolution kernel with a size of 1 × 1 and a stride of 2 is used instead of the pooling layer to achieve the proportional reduction of the feature map size, so that the feature maps output by conv3, conv4, conv5, conv6 is 1/4, 1/8, 1/16, and 1/32 of the input image, respectively.

(2) $$\left\{\begin{array}{c} Size_{conv3}=\frac{Size_{input}}{4}\\ Size_{conv4}=\frac{Size_{input}}{8}\\ Size_{conv5}=\frac{Size_{input}}{16}\\ Size_{conv6}=\frac{Size_{input}}{32} \end{array}\right.$$

(2) The output feature maps of the convolution modules conv3_3, conv4_3, and conv5_4 are down-sampled, and the average pooling layers with kernel sizes of 8 × 8, 4 × 4, and 2 × 2 are used, so that their respective output feature maps are consistent with the feature map output by conv6_2

(3) $$\left\{\begin{array}{c} f_{c1}=Pool_{Avg}^{8\times 8}\left(f_{conv3\_3}\right)\\ f_{c2}=Pool_{Avg}^{4\times 4}\left(f_{conv4\_3}\right)\\ f_{c3}=Pool_{Avg}^{2\times 2}\left(f_{conv5\_4}\right)\\ f_{c4}=f_{con6\_2} \end{array}\right.$$

where $f_{conv3\_3},f_{conv4\_3},f_{conv5\_4}\;\mathrm{and}\;f_{conv6\_2}$ represent the output feature maps of conv3_3, conv4_3, conv5_4 and conv6_2, respectively, $f_{c1}$, $f_{c2},\;f_{c3}\;\mathrm{and}\;f_{c4}\;$, respectively, represent the four input feature maps of the concatenation layer.

(3) The above four feature maps are combined and connected to form the final output feature map, the formula is as follows:

(4) $$f_{concat}=concat\left(f_{c1},f_{c2},f_{c3},f_{c4}\right)$$

where $f_{concat}$ represents the feature map output by the concatenation layer. In this way, the final output features include small-local concrete features and macro-global abstract features.

It is worth mentioning that the architecture design of MSF-Net follows many experiences and guidelines for the design of CNNs, as follows:

(1) Avoiding expression bottlenecks in the early stage of feature extraction. That is, the information flow should avoid highly compressed convolution layers in the forward propagation process, and the width and height of the feature map should be gradually reduced in an orderly manner, especially for surface defect datasets with subtle defect features, it is not wise to compress the feature map too early. Therefore, the convolutional layer conv1_1 (size 3 × 3, stride 1) and pooling layer pool1_1 (size 3 × 3, stride 2) are concatenated to slow down the reduction speed of the feature map.
(2) In the middle and late stages of feature extraction in CNNs, the width and depth of CNNs should be balanced as much as possible. That is, as the CNN deepens, the feature map gradually shrinks, and the output matrix dimension of each convolutional layer should gradually increase. Therefore, the number of modules, the feature map sizes, and the number of channels of the three module chains of conv 4, conv 5, and conv 6 are designed with full reference to Inception V1 [29] and Inception V3 [32] to improve the rationality of CNN’s evolution.
(3) Average pooling layer is used to replace the fully connected layer, which can greatly reduce the number of parameters and save calculation costs. Specifically, MSF-Net uses an average pooling layer with a kernel size of 7 × 7 and a strider of 1 to replace the fully connected layer. It can be calculated from Table 4 that this adjustment can reduce 180,635,529 parameters.
(4) The residual shortcut connections are used to effectively accelerate training and promote CNN’s convergence. Specially, almost every convolution module in MSF-Net, except conv1_1, uses a shortcut connection, which effectively avoids the problem of gradient disappearance and speeds up training.

## 4. Experimental Results and Analysis

### 4.1. Experimental Setup

The performance indicators and parameters of the experimental platform are shown in Table 5.

### 4.2. CNNs for Comparison

Inception v3 and ResNet-50 are used as the comparison CNNs for the following reasons:

(1) Inception v3 and ResNet-50 are closer to MSF-Net in terms of the number of convolutional layers and parameters, as shown in Table 6, which makes the comparison of experimental results fairer.
(2) MSF-Net is deeply influenced by GoogLeNet v3 in terms of the design of feature extraction modules, the number of modules, and the width and depth of CNN. Therefore, comparing MSF-Net with Inception v3 can more accurately assess the impact of CNN’s structure on classification performance.
(3) Except conv1_1, all feature extraction modules of MSF-Net use residual shortcut connections.

Therefore, it is also essential to conduct comparative experiments with ResNet-50, which is similar in size.

### 4.3. Training Efficiency Evaluation of CNNs

**Figure 13:**
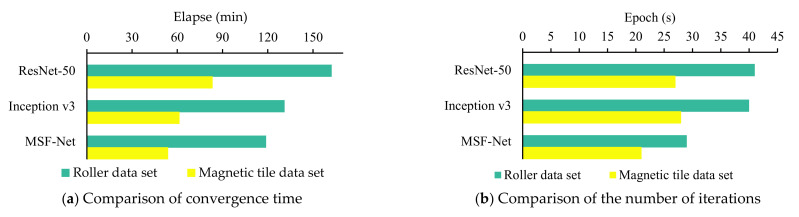
Comparison of the time and the number of epochs required for three CNNs to reach convergence.

Figure 13 shows the comparison of the time and number of iterations required to reach convergence when the three CNN models are trained on two multi-scale surface defect data sets. It can be seen from Figure 13a that MSF-Net achieves convergence in the shortest time on the training performance of the two multi-scale surface defect data sets. Specifically, the convergence time of MSF-Net is shortened by at least 25% compared with that of ResNet-50, and it is also shortened by nearly 10% compared with that of Inception v3. Figure 13b shows the comparison of the number of epochs for the three models to reach convergence. Through the comparative analysis of three CNNs, it can be seen that the efficient training performance of MSF-Net mainly comes from the following two aspects:

(1) Compared with ResNet-50 and Inception v3, MSF-Net’s parameters is reduced by more than 54%, which greatly reduces the computational cost, the most intuitive manifestation is that the number of epochs required for MSF-Net to achieve convergence is greatly reduced;
(2) Compared with the Inception modules fully used by Inception v3, MSF-Net uses the optimized CReLU module in the early stage of feature extraction, the optimized CReLU module has outstanding performance in reducing computational costs, which effectively shortens the time consumption of forward back propagation and improves the training performance of MSF-Net.

### 4.4. Classification Performance Evaluation on Two Multi-Scale Defect Data Sets

**Figure 14:**
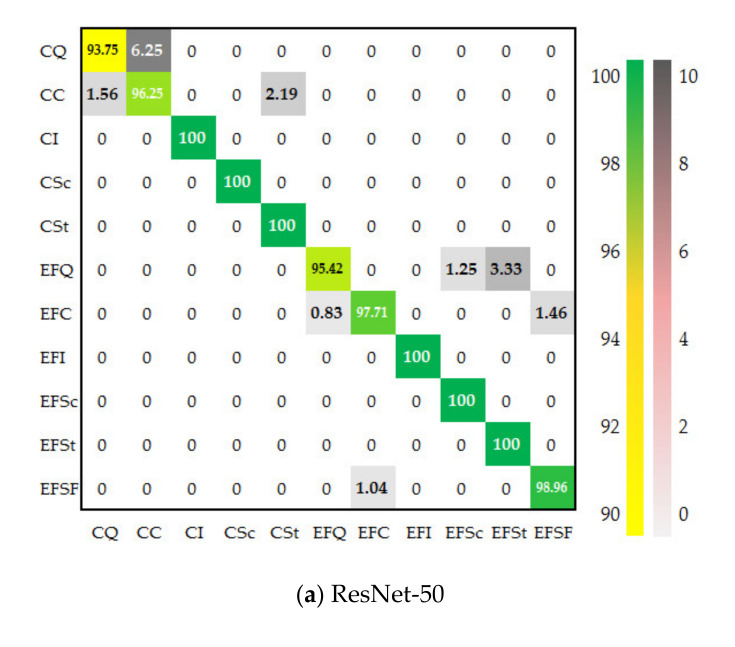

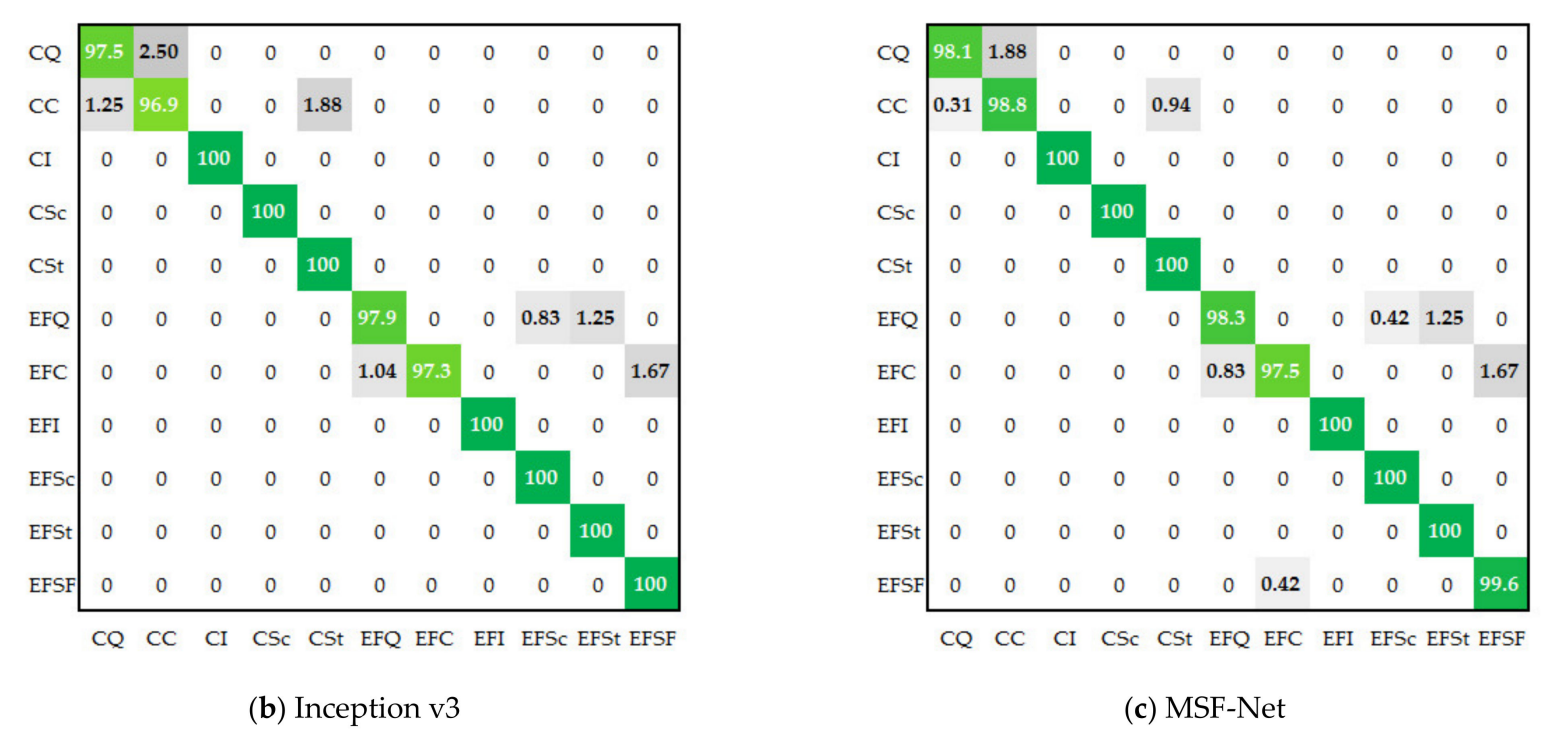
Confusion matrix of three CNNs on the surface defect data set of roller (unit: %).

Table 7 and Figure 14 respectively show the classification performance of ResNet-50, Inception v3, and MSF-Net on the test of the surface defect data set of roller. It can be seen from Table 7 that the three CNNs have excellent performance in the defect categories of CI, CSs, CSt, EFI, EFSc, and EFSt, with an accuracy rate of 100%. In the remaining five categories, CQ and EFQ are non-defective samples; CC and EFC are small-size defects, and the appearance of the samples is very close to CQ and EFQ respectively, EFSF represents larger defect samples. Therefore, the roller surface defect data set is very suitable for the evaluation and verification of multi-scale feature learning capabilities of CNNs. ResNet-50 and Inception v3 achieved the highest recall rates in the EFC and EFSF defect categories, respectively, while MSF-Net performed better than the other two CNNs in the CC defect category. In addition, MSF-Net has an outstanding performance in the accuracy rate of CQ and EFQ, which has important application value in actual production. Overall, the average recall rate of MSF-Net on the roller defect set is 99.29%, while the recall rates of ResNet-50 and Inception v3 are 98.44% and 99.06%, respectively; at the same time, MSF-Net has the smallest standard deviation in recall rate, showing a more balanced expression and learning ability for defects of different scales. Similarly, compared with ResNet-50 and Inception v3, MSF-Net also achieves the best performance in precision, micro-F1 and macro-F1 indicators, which verified its superiority.

**Figure 15:**
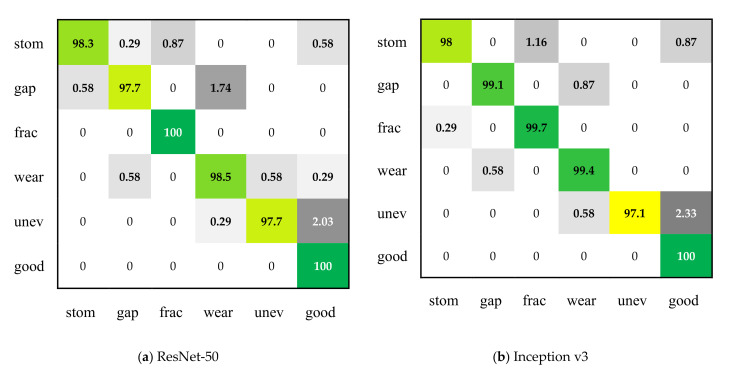

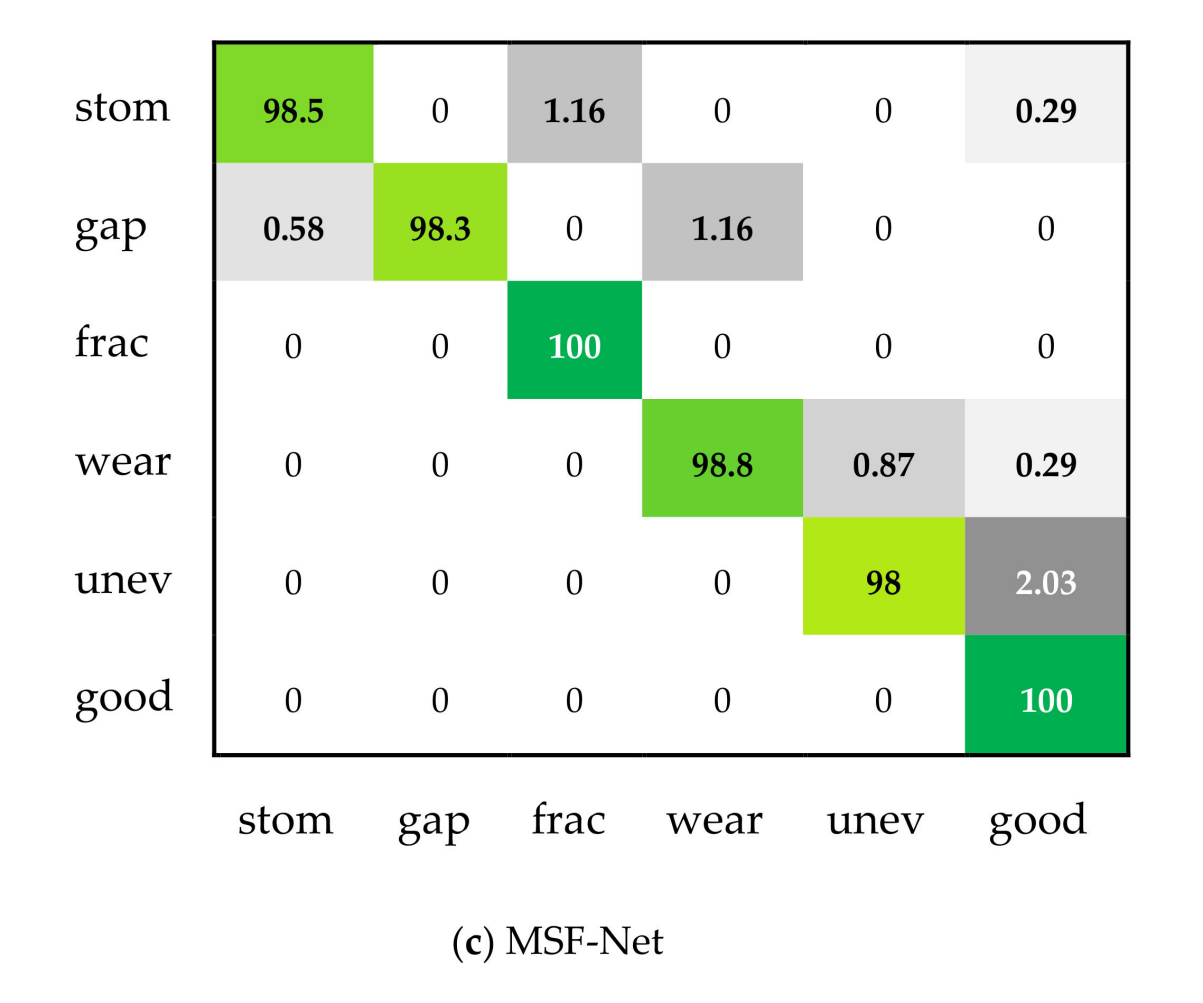
Confusion matrix of three CNNs on the surface defect data set of magnetic tile (Abbreviation description—stom: stomatal; frac: fracture; unev: uneven).

Table 8 and Figure 15 show the classification performance of ResNet-50, Inception v3, and MSF-Net on the test set of surface defect data set of magnetic tile. It can be seen from Table 8 that the three CNNs have excellent performance in the categories of fracture defects and good products. MSF-Net has the highest accuracy rate for stomatal and uneven defects, and Inception v3 has the best performance on the gap and wear defect categories. In general, the average recall rate of MSF-Net on the magnetic tile defect set reached 98.93%, while the recall rate of ResNet-50 and Inception v3 were 98.69% and 98.89% respectively; at the same time, among the three CNNs, MSF-Net has the smallest standard deviation in recall rate. Similarly, MSF-Net performs better on precision, micro-F1 and macro-F1 than ResNet-50 and Inception v3.

## 5. Conclusions

Aiming at the problem that the commonly used CNNs are not ideal for detecting small and local defects on the products’ surface, the multi-scale feature extraction mechanism involved in several mainstream CNNs in the current deep learning field is analyzed, and a multi-scale feature learning network based on dual module feature extractor is proposed, named MSF-Net, the design of the dual module feature extractor, the specific architecture and parameters of MSF-Net, and the optimization to improve training efficiency are introduced in detail. The proposed MSF-Net was trained and tested on two multi-scale surface defect data sets, which verified the advancement and effectiveness in multi-scale defect detection. Future work will focus on exploring the generalization of MSF-Net in more research fields, such as radar image classification and remote sensing image classification.

## Figures and Tables

**Table 1:** The receptive fields of the last convolution layer of feature maps of AlexNet and VGG-16.

CNNs	Feature Maps of the Last Convolution Layer	Receptive Field
AlexNet	pool5	195 × 195
VGG-16	pool5	212 × 212

**Table 2:** The maximum and minimum values of the receptive field of the last convolution layer of feature maps of GoogLeNet and ResNet-18.

CNNs	Feature Map of the Last Convolutional Layer	Minimal Receptive Field	Maximum Receptive Field
GoogLeNet	pool5/7 × 7_s1	267 × 267	907 × 907
ResNet-18	pool5	203 × 203	627 × 627

**Table 3:** The sample examples and numbers of each category in the roller surface defect data set.

Category Name	EFQ *	EFC	EFI	EFSc	EFSt	EFSF
Number of samples	1500	470	70	160	90	220
Sample example	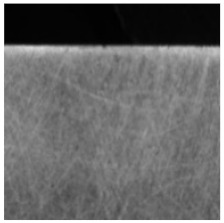	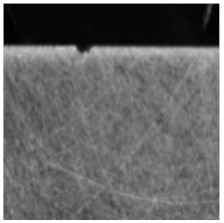	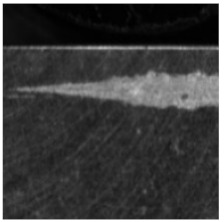	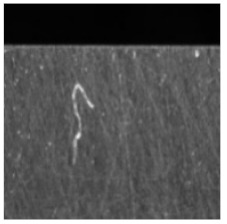	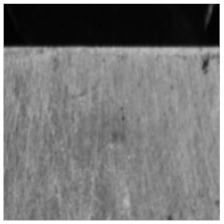	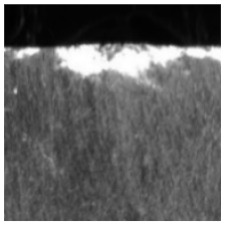
Category name	CQ	CC	CI	CSc	CSt	
Number of samples	1000	350	155	30	105	
Sample example	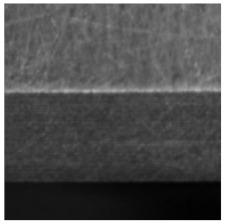	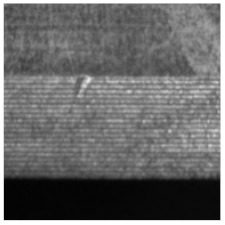	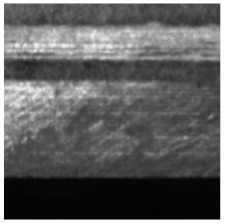	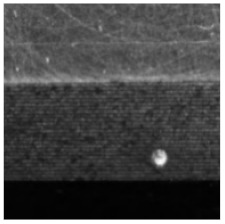	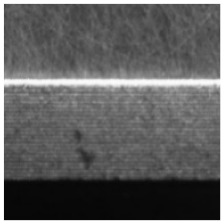	

* EFQ: end-face qualified; EFC: end-face cracks; EFI: end-face indentations; EFSc: end-face scratches; EFSt: end-face stains EFSF: end-face serious fracture CQ: chamfer qualified CC: chamfer cracks CI: chamfer indentations CSs: chamfer scratches CSt: chamfer stains.

**Table 4:** The parameters of MSF-Net.

Layername	Type	Output Dimension	Depth	CReLU Output	Inception Output					Parameters
				#1×1-3×3-1×1	Pool Proj	#1×1	#3×3	#5×5	#1×1 out	
*conv1_1*	3 × 3 CReLU	224 × 224 × 32	1	NA-16-NA	/					480
*pool1_1*	3 × 3 Max pool.	112 × 112 × 32	0	/						/
*conv2_1*	3 × 3 CReLU	112 × 112 × 64	3	24-24-64	/					13,440
*conv2_2*	3 × 3 CReLU	112 × 112 × 64	3	24-24-64						13,440
*conv2_3*	3 × 3 CReLU	112 × 112 × 64	3	24-24-64						13,440
*conv3_1*	3 × 3 CReLU	56 × 56 × 128	3	48-48-128	/					53,760
*conv3_2*	3 × 3 CReLU	56 × 56 × 128	3	48-48-128						53,760
*conv3_3*	3 × 3 CReLU	56 × 56 × 128	3	48-48-128						53,760
*conv4_1*	Inception	28 × 28 × 256	4	/	32	64	96-128	16-32-32	256	322,560
*conv4_2*	Inception	28 × 28 × 256	4		32	64	96-128	16-32-32	256	322,560
*conv4_3*	Inception	28 × 28 × 256	4		32	64	96-128	16-32-32	256	322,560
*conv5_1*	Inception	14 × 14 × 512	4	/	64	128	128-192	32-96-96	512	1,040,384
*conv5_2*	Inception	14 × 14 × 512	4		64	128	128-192	32-96-96	512	1,040,384
*conv5_3*	Inception	14 × 14 × 512	4		64	128	128-192	32-96-96	512	1,040,384
*conv5_4*	Inception	14 × 14 × 512	4		64	128	128-192	32-96-96	512	1,040,384
*conv6_1*	Inception	7 × 7 × 1024	4	/	128	256	160-320	32-128-128	1024	3,010,560
*conv6-2*	Inception	7 × 7 × 1024	4		128	256	160-320	32-128-128	1024	3,010,560
*concat*	Concatenation	7 × 7 × 1920	0	/	/					/
*avg pool*	7 × 7 Avg pool.	1 × 1 × 1920	0							
*linear*	Inner product	1 × 1 × $N_{Class}$	1							19,200
Total			47							11,371,616

**Table 5:** Parameters of the experimental platform.

CPU	Intel E3-1230 V2*2 (3.30 GHz)	RAM	16 GB DDR3	GPU	NVIDIA GTX-1080Ti
OS	Ubuntu 16.04 LTS	Software	Visual Studio Code with Python 2.7		

**Table 6:** Comparison of the number of convolutional layers and parameters of three CNNs.

CNNs	Convolution Layers	Parameters
Inception v3	48	24,734,048
ResNet-50	50	~25.5 × 10^6^
MSF-Net	56	11,371,616

**Table 7:** Classification performance of three CNNs on the surface defect data set of roller (%).

	CQ	CC	CI	CSc	CSt		Precision	micro-F1
*ResNet-50*	93.75	96.25	100.00	100.00	100.00		98.46 ± 0.017	98.381
*Inception v3*	97.50	96.88	100.00	100.00	100.00		99.09 ± 0.008	99.055
*MSF-Net*	98.13	98.75	100.00	100.00	100.00		99.29 ± 0.006	99.301
	**EFQ**	**EFC**	**EFI**	**EFSc**	**EFSt**	**EFSF**	**Recall**	**macro-F1**
*ResNet-50*	95.42	97.71	100.00	100.00	100.00	98.96	98.44 ± 0.022	98.367
*Inception v3*	97.92	97.29	100.00	100.00	100.00	99.79	99.06 ± 0.013	99.051
*MSF-Net*	98.33	97.50	100.00	100.00	100.00	99.58	99.29 ± 0.009	99.298

**Table 8:** Classification performance of three CNNs on the surface defect data set of magnetic tile (%).

	Stomatal	Gap	Fracture	Precision	Micro-F1
*ResNet-50*	98.26%	97.67%	100.00%	98.70 ± 0.008	98.70
*Inception v3*	97.97%	99.13%	99.71%	98.90 ± 0.010	98.90
*MSF-Net*	98.55%	98.26%	100.00%	98.94 ± 0.007	98.94
	**Wear**	**Uneven**	**Good**	**Recall**	**Macro-F1**
*ResNet-50*	98.55%	97.67%	100.00%	98.69 ± 0.009	98.69
*Inception v3*	99.42%	97.09%	100.00%	98.89 ± 0.010	98.89
*MSF-Net*	98.84%	97.97%	100.00%	98.93 ± 0.008	98.93

## Data Availability

Not applicable.
